# A Low-Carbohydrate Diet Improves Glucose Metabolism in Lean Insulinopenic Akita Mice Along With Sodium-Glucose Cotransporter 2 Inhibitor

**DOI:** 10.3389/fendo.2020.601594

**Published:** 2020-12-11

**Authors:** Yukihiro Fujita, Kuralay K. Atageldiyeva, Yasutaka Takeda, Tsuyoshi Yanagimachi, Yuichi Makino, Masakazu Haneda

**Affiliations:** ^1^ Division of Metabolism and Biosystemic Science, Department of Internal Medicine, Asahikawa Medical University, Asahikawa, Japan; ^2^ Division of Diabetology, Endocrinology and Nephrology, Department of Internal Medicine, Shiga University of Medical Science, Otsu, Japan; ^3^ School of Medicine, Nazarbayev University, Nur-Sultan City, Kazakhstan; ^4^ Integrated Medical Education Center, Asahikawa Medical University, Asahikawa, Japan

**Keywords:** low-carbohydrate diet, sodium-glucose cotransporter 2 inhibitor, ketogenesis, islet morphology, Akita mice

## Abstract

**Objective:**

A low-carbohydrate diet (LC) can be beneficial to obese subjects with type2 diabetes mellitus (T2DM). Sodium-glucose cotransporter 2 inhibitor (SGLT2i) presents prompt glucose-lowering effects in subjects with T2DM. We investigated how LC and SGLT2i could similarly or differently influence on the metabolic changes, including glucose, lipid, and ketone metabolism in lean insulinopenic Akita mice. We also examined the impacts of the combination.

**Methods:**

Male Akita mice were fed ad libitum normal-carbohydrate diet (NC) as a control or low-carbohydrate diet (LC) as an intervention for 8 weeks with or without SGLT2i treatment. Body weight and casual bold glucose levels were monitored during the study, in addition to measuring TG, NEFA, and ketone levels. We quantified gene expressions involved in gluconeogenesis, lipid metabolism and ketogenesis in the liver and the kidney. We also investigated the immunostaining analysis of pancreatic islets to assess the effect of islet protection.

**Results:**

Both LC and SGLT2i treatment reduced chronic hyperglycemia. Moreover, the combination therapy additionally ameliorated glycemic levels and preserved the islet morphology in part. LC but not SGLT2i increased body weight accompanied by epididymal fat accumulation. In contrast, SGLT2i, not LC potentiated four-fold ketone production with higher ketogenic gene expression, in comparison with the non-treated Akita mice. Besides, the combination did not enhance further ketone production compared to the SGLT2i alone.

**Conclusions:**

Our results indicated that both LC and SGLT2i reduced chronic hyperglycemia, and the combination presented synergistic favorable effects concomitantly with amelioration of islet morphology, while the combination did not enhance further ketosis in Akita mice.

## Introduction

It is widely appreciated that nutritional intervention is a major approach as the treatment for diabetes to maintain adequate body weight, especially for obese subjects with insulin resistance. In nutritional interventions, total calorie restriction is conventionally practiced, but nutrition-oriented restrictions such as a low-carbohydrate diet (LC), a low-fat diet or Mediterranean diet are alternatively preferred, especially for obese type 2 diabetes mellitus (T2DM) with insulin resistance (1, 2). LC is supposed to be beneficial to obese subjects with T2DM but may be controversial to T2DM subjects with impaired insulin secretion or T1DM subjects. Indeed, some reports indicated that LC induced life-threatening ketoacidosis in subjects with T1DM, who intentionally reduced daily dosage of insulin injection or developed to euglycemic diabetic ketoacidosis (3, 4).

Sodium-glucose cotransporter 2 inhibitor (SGLT2i) presents prompt glucose-lowering effects along with body weight loss in the over-weighted subjects with type 2 diabetes mellitus (T2DM) (5). Recent randomized clinical trials revealed that SGLT2i could be a promising agent preventing cardiovascular events, progression of diabetic kidney disease, and decreasing mortal events in T2DM (6, 7). SGLT2i exerts the therapeutic activity by facilitating glucose excretion through the kidney, independently on insulin actions. The mechanism beyond the glycemic improvement, such as the increment of ketone bodies and hemodynamic changes can trigger preferable effects on the prevention of cardiovascular and renal impairment (8, 9). Clinical trials revealed that SGLT2i could improve glycemic control and reduced the dosage of insulin, even in subjects with type 1 diabetes mellitus (T1DM) (10). Thus, SGLT2i is a potential tool to treat T1DM with insulin injection, adversely with a slightly higher risk of ketoacidosis (10).

We have previously examined how LC and SGLT2i can impact on metabolic changes, especially gluconeogenesis and glycogen storage using non-diabetic animals (11). SGLT2i, but not LC reduced glucose excursion after an oral glucose challenge test. LC increased body weight, epidydimal fat mass, and developed insulin resistance, while both the LC and SGLT2i treatment enhanced food intake on a calorie basis. Gluconeogenic glucose-6-phosphatase (*G6pc*), phosphoenolpyruvate carboxyl kinase (*Pck*), and fructose-1,6-bisphosphatase (*Fbp*) were simultaneously increased in the liver by LC treatment, as well as enhanced in the kidney by SGLT2i treatment. Not LC but SGLT2i treated group showed markedly lower glycogen content in the liver. Thus, we concluded that LC and SGLT2i may have different metabolic effects on the kidney and the liver in the non-diabetic animals (11).

Few studies have been conducted to compare LC and SGLT2 in diabetic states, especially with insulin insufficiency or T1DM. Therefore, we investigated how LC, SGLT2i could similarly or differently influence the metabolic changes, including glucose, lipid, and ketone metabolism in the liver and the kidney in lean insulinopenic Akita mice and examine the impacts of the combination of LC and SGLT2i treatments.

## Material and Methods

### Ethical Approval of the Study Protocol

All procedures related to animals were carried out in strict accordance with the Guidelines for Animal Experimentation at Asahikawa Medical University (Asahikawa, Japan), Animal Protection and Management Law set by the Japanese Government. The experimental protocol was approved by the Asahikawa Medical University Animal Research Committee (No15070, No14063, and No16129).

### Experimental Protocol

Six-week-old male diabetic Akita mice (AKITA/Slc) and non‐diabetic C57BL/6 mice (C57BL/6J) were purchased from Charles River Laboratories Japan Inc. (Yokohama, Japan). All mice were maintained under a 12‐h light-dark cycle with free access to food and water.

The study protocol is shown in **Supplemental Figure 1**. Briefly, after two days of acclimatization, mice were fed ad libitum normal-carbohydrate diet (NC) as a control or low-carbohydrate diet (LC) as an intervention for 8 weeks with or without SGLT2i treatment. Akita mice were randomized into four groups (each n = 6): the LC group was fed with LC alone, the NC+Ipra group was treated with an SGLT2i ipragliflozin alone and fed with NC, the LC+Ipra combined group was fed with LC and treated with Ipragliflozin, and the NC group was a non-treated control, respectively. The NN group was set as a non-diabetic control group fed with NC. NC and LC consist of the identical formulas which we utilized in our previous study (11). Briefly, NC consists of carbohydrate: protein: fat (C: P: F) = 68:21:12% kJ and LC consists of C: P: F = 16:40:44% kJ as shown in **Table 1**. Ipragliflozin was kindly provided by Astellas Pharma Inc. (Ibaraki, Japan). Ipragliflozin (3 mg/kg body weight) or NaCl (154 mmol/l) solution was administered daily by oral gavage between 4 and 6 pm as described previously (11). Body weights and non-fasted blood glucose levels were measured with an enzymatic colorimetric assay (LabAssay Glucose Kit; Wako Pure Chemical Industries, Ltd., Osaka, Japan) at the start of the study and twice a week thereafter until day 56. Food intake was measured every day during the experiment. For fasting studies, mice were fasted overnight (18 h) before analysis.

**Table 1:** Experimental Diets.

Product #	D10001 (NC)		D14012301 (LC)	
	gm%	kcal%	gm%	kcal%
Protein	20	21	47	40
Carbohydrate	66	68	19	16
Fat	5	12	23	44
Total		100		100
**kcal/gm**	3.9		4.7	
**Ingredient**	**Gm**	**kcal**	**Gm**	**Kcal**
Casein	200	800	384	1,536
DL-Methionine	3	12	6	24
Corn Starch	150	600	50	200
Maltodextrine	0	0	100	400
Sucrose	500	2,000	0	0
Cellulose, BW200	50	0	50	0
Corn oil	50	450	50	450
Lard	0	0	139	1251
Mineral Mix S10001	35	0	35	0
Vitamin Mix V10001	10	40	10	40
Choline Bitertrate	2	0	2	0
FD&C Red Dye #40	0	0	0.05	0
Total	1000	3902	826.05	3901

### Glucose-Stimulated Insulin Secretion

We examined glucose-stimulated insulin secretion at day 53 of the study. Glucose solution (25 (wt/v) %) was orally administered (2mg/g body weight) by gavage after 18-h fasting. Blood was obtained from the tail vein at 0 and 15 min after gavage. Blood samples were collected into heparinized tubes at 0 and 15 min and centrifuged for the analysis of glucose levels and glucose-stimulated blood insulin levels.

### Plasma Biochemical Analysis

Twenty-four-hour urine was collected in a metabolic cage at day 42. Urinary glucose levels were measured using an enzymatic colorimetric assay (LabAssay Glucose Kit; Wako Pure Chemical Industries, Ltd., Osaka, Japan). Non-esterified fatty acid (NEFA), triglycerides (TG), 3-hydroxybutyrate (3-OHBA) levels were measured at day 39 by enzymatic colorimetric assays (LabAssay NEFA Kit and LabAssay Triglyceride Kit; Wako Pure Chemical Industries, Ltd., Osaka, Japan; Ketorex Kit; Sanwa Kagaku Kenkyusho Co.Ltd., Nagoya, Japan) after overnight fasting. Plasma insulin was measured using a commercial ELISA kit (Ultra Sensitive Mouse Insulin ELISA Kit; Morinaga Institute of Biological Science Inc. Yokohama, Japan). Glycated hemoglobin (HbA1c) levels were measured using DCA Vantage Analyzer (Siemens, Munich, Germany) at day 53.

### Measurement of Urinary Albumin, Creatinine, and Glucose

Albuminuria was evaluated using an enzyme immunoassay Kit from Exocell (Philadelphia, USA). Creatinine concentration was measured using a picric acid-based assay kit from Wako Pure Chemical Industries, Ltd., (Osaka, Japan). Urinary glucose levels were measured using an enzymatic colorimetric assay (LabAssay Glucose Kit; Wako Pure Chemical Industries, Ltd., Osaka, Japan).

### Tissue Collection

At day 56, the last day of the study, non-fasted mice were sacrificed under whole body inhalable anesthesia (isoflurane 2%, Abbott Japan Co. Ltd., Tokyo, Japan). The Kidney and the liver were rapidly removed, parts of those were flash-frozen in liquid nitrogen for RNA extraction and tissue glycogen content, and the rest part was fixed overnight for histological analysis. Epididymal fat pads were dissected from each animal and weighed to evaluate visceral fat accumulation.

### Quantitative Analysis of mRNA Expression in the Kidney and the Liver

Total RNA was isolated from the kidney and the liver tissues using Trizol Reagent (Life Technologies, CA, USA). cDNA was synthesized using a SuperScript First-Strand Synthesis System for RT-PCR (Invitrogen, CA, USA). Quantitative RT-PCR was performed with TaqMan® Universal PCR Master Mix (Applied Biosystems) using a 7300 Real-Time PCR system (Applied Biosystems, Waltham, Massachusetts, USA). Gene expression was determined using primers and probes (Taqman Gene Expression Assays) purchased from Applied Biosystems (San Francisco, CA USA). Gene expression relative to the housekeeping gene *Rpl37A* was quantified by the 2 –ΔΔCt method (11).

### Tissue Glycogen Content in the Liver

After 100 mg of liver tissue was digested overnight, tissue glycogen content was measured by colorimetric assay using the Glycogen Assay Kit II (#Ab169558, Abcam, Cambridge, UK) (11).

### Immunostaining

Immunostaining was performed as we previously reported (12, 13). Briefly, the pancreas tissues were collected at day 56 were fixed in 4 (wt/v) % paraformaldehyde in PBS overnight at 4°C and then embedded in paraffin blocks after an ethanol wash. Embedded tissue was sliced into 3 μm thick sections and deparaffinized with a series of xylene and ethanol. We performed antigen retrieval with sodium citrate buffer (10 mmol/l sodium citrate containing 0.05 (wt/v) % Tween 20, pH 6.0) followed by microwave heating and treatment of sections with a reagent for blocking non-specific background staining (Dako Japan, Tokyo, Japan). Slides were incubated with primary antibody overnight at 4°C, and, they were incubated with secondary antibody for 2 h at room temperature in the shade after they were washed three times. They were then washed and finally mounted in aqueous medium with DAPI (Vectashield Mounting Medium with DAPI; Vector Laboratories, Burlingame, CA, USA). Slides were observed under fluorescence microscopy (BZ-8100; Keyence, Osaka, Japan), and digital images were collected. We used a guinea pig antibody against insulin (4011-01F, Millipore, Billerica, MA, USA), a mouse antibody against glucagon (G2654, Sigma-Aldrich Japan, Tokyo, Japan) as primary antibodies. Alexa Fluor 488 anti-guinea pig IgG, Alexa Fluor 488 anti-rabbit IgG, Alexa Fluor 594 (Thermo Fisher Scientific, Tokyo, Japan) were used as secondary antibodies.

### Statistical Analysis

All data are expressed as mean ± SEM from repeated experiments. The statistical analyses were determined by one-way ANOVA, Student’s T-test or two-way ANOVA. For the ANOVA procedures, Bonferroni tests were used to establish differences between groups. Statistical significance was set at p < 0.05.

## Results

### LC and SGLT2i Additionally Ameliorated the Development of Chronic Hyperglycemia in Akita Mice

**Figure 1:**
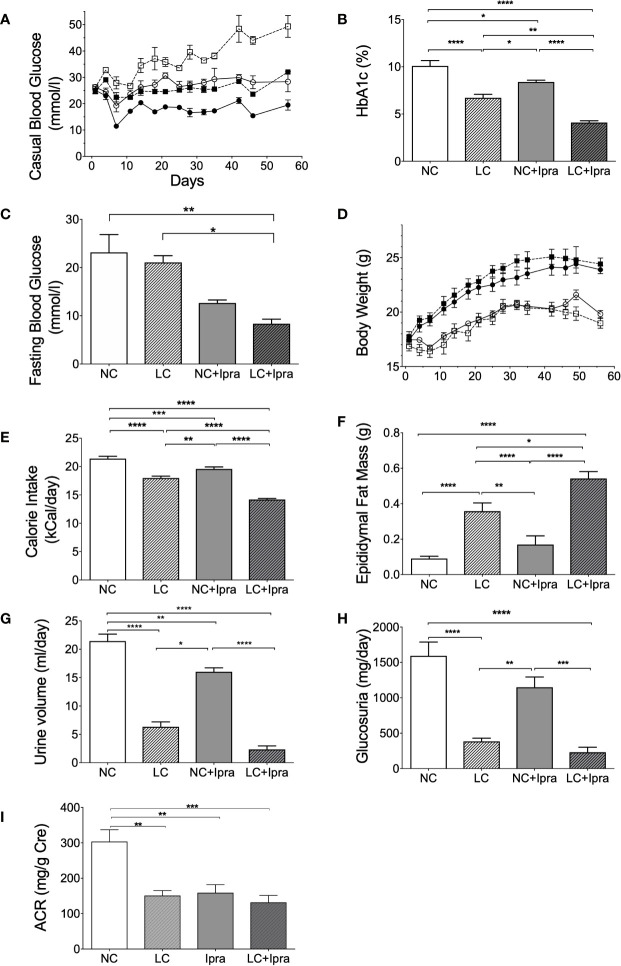
The effect of low-carbohydrate diet (LC), sodium-glucose cotransporter 2 inhibitor (SGLT2i) and, the combination on metabolic parameters. **(A)** Casual blood glucose [normal-carbohydrate diet (NC) vs LC p < 0.001; NC vs NC+Ipra p < 0.005; NC vs LC+Ipra p < 0.001; LC vs NC+Ipra ns; LC vs LC+Ipra p < 0.01; NC+Ipra vs LC+Ipra <0.01]. **(B)** HbA1c, **(C)** fasting blood glucose at day 53. **(D)** Body weight (NC vs LC p < 0.001; NC vs NC+Ipra ns; NC vs LC+Ipra p < 0.005; LC vs NC+Ipra p < 0.001; LC vs LC+Ipra ns; NC+Ipra vs LC+Ipra < 0.01), **(E)** average mean daily calorie intake per mouse, **(F)** epididymal fat mass. **(G)** Urine volume per day at day 42, **(H)** daily glucosuria. *p < 0.05, **p < 0.01, ****p < 0.0005. **(I)** urinary albumin creatinine ratio (ACR). **p < 0.01, ***p < 0.001. Data are presented as means ± SEM. n=6, each group. White squares, NC; black squares, LC; white circles, NC+Ipra; black circles, LC+Ipra **(A, C)**. White bars, NC; hatched white bars, LC; grey bars, NC+Ipra; hatched gray bars, LC+Ipra **(B, D–I)**.

We monitored the metabolic parameters in Akita mice during the 8-week treatment. Both SGLT2i and LC treatment prevented the development of chronic hyperglycemia in Akita mice, compared to the non-treated control NC (**Figure 1A**). Further, the combination therapy (LC+Ipra) significantly ameliorated casual glycemic levels compared to LC or NC+Ipra. Similarly, we observed that the combination therapy more effectively lowered HbA1c levels than LC alone or NC+Ipra (**Figure 1B**). The combination therapy decreased fasting glucose levels compared to the non-treated control, while LC alone did not (**Figure 1C**).

LC, but not SGLT2i, significantly increased body weight in lean Akita mice, accompanied by visceral fat accumulation during the 8-week treatment (**Figures 1D, F**). In the assessment of calorie intake based on food consumption, both LC and SGLT2i treatment significantly reduced and the combination further decreased calorie intake in Akita mice (**Figure 1E**). LC, SGLT2i or the combination treatment (LC+Ipra) decreased daily urinary volume (**Figure 1G**). In contrast, LC, but not SGLT2i, significantly decreased urinary glucose disposal (**Figure 1H**).

We investigated the effects of the treatments to diabetic kidney disease. LC, SGLT2i, and the combination treatment significantly reduced urinary albumin creatinine ratio, compared to the non-treated control NC (**Figure 1I**).

### The Combination Therapy Partially Preserved the Islet Architecture

**Figure 2:**
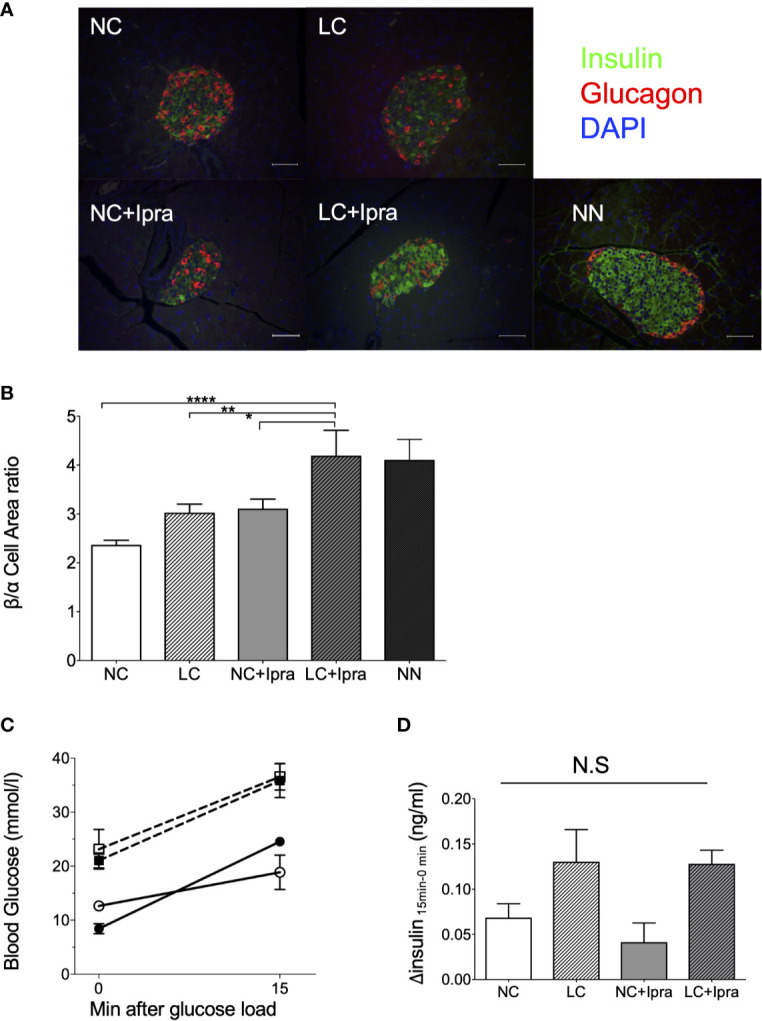
Islet morphology and beta-cell function. **(A)** Immunofluorescent images in the islets. A white bar indicates 50 µm. Insulin: green, glucagon: red, DAPI: blue. x200 magnification. **(B)** Beta-cell/alpha cell area ratio. From three mice, a of total 34–57 islets were counted. Data are presented as means ± SEM. n=6, each group. *p < 0.05, **p < 0.01, ****p < 0.0001. **(C)** Glycemic change during oral glucose load. **(D)** Glucose-stimulated insulin secretion by 2 g/Kg body weight oral glucose load. Delta insulin levels (insulin at 15 min minus insulin at 0 min) are shown. White bars, normal-carbohydrate diet (NC); hatched white bars, low-carbohydrate diet (LC); grey bars, NC+Ipra; hatched gray bars, LC+Ipra **(B, D)**. White squares, NC; black squares, LC; white circles, NC+Ipra; black circles, LC+Ipra **(C)**.

Next, we examined whether LC, SGLT2i, or the combination can ameliorate beta-cell function and islet architecture in the insulinopenic mice. We observed the combination therapy significantly raised beta/alpha cell area ratio, while severe destruction of beta-cells and expansion of alpha-cell area was noted in the non-treated Akita mice, suggesting that combination therapy preserved the islet morphology in part (**Figures 2A, B**). The SGLT2i lowered glycemic levels 15 min after glucose load, although treatments failed to improve glucose-stimulated insulin secretion in Akita mice (**Figures 2C, D**).

### Gene Expression of Gluconeogenesis in the Liver and the Kidney of Akita Mice

**Figure 3:**
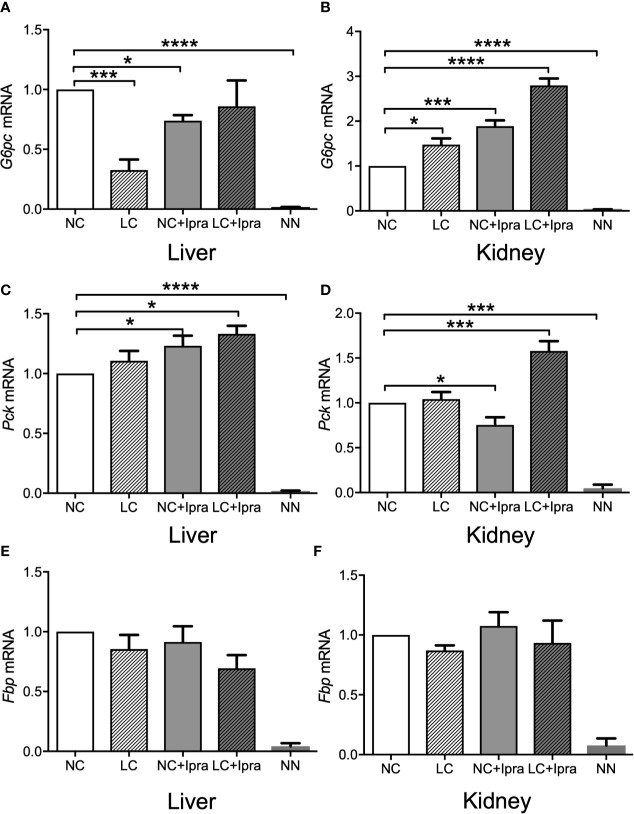
Glucogenic gene expressions in the liver and the kidney. **(A, C, E)** present expression in the liver and **(B, D, F)** present expression in the kidney. **(A, B)** *G6pc mRNA*, **(C, D)** *Pck mRNA*, **(E, F)** *Fbp mRNA*. Data are presented as means ± SEM. n=6, each group. *p < 0.05, ***p < 0.001, ****p < 0.0001. White bars, normal-carbohydrate diet (NC); hatched white bars, low-carbohydrate diet (LC); grey bars, NC+Ipra; hatched gray bars, LC+Ipra. NN: non-diabetic mouse.

Our previous report demonstrated that gluconeogenic *G6PC*, *Pck*, and *Fbp* gene expressions were simultaneously increased in the kidney by SGLT2i treatment, as well as enhanced in the liver by LC treatment in the non-diabetic mice. We examined these gene expressions in the liver and the kidney of diabetic Akita mice and investigated whether these treatments can influence the gene expressions. The gluconeogenic *G6PC*, *Pck*, and *Fbp* gene expressions were markedly increased both in the liver and in the kidney of Akita mice compared to those of the non-diabetic mice (**Figures 3A–F**). LC and SGLT2i treatment significantly decreased *G6PC mRNA* expression in the liver, but increased in the kidney (NC vs LC, NC vs NC+Ipra). The combination increased *Pck* mRNA expression both in the liver and the kidney. In contrast, those treatments did not change *Fbp* mRNA expression in either the liver or the kidney.

### SGLT2i, But Not LC Treatment, Induced Ketogenesis in Akita Mice

**Figure 4:**
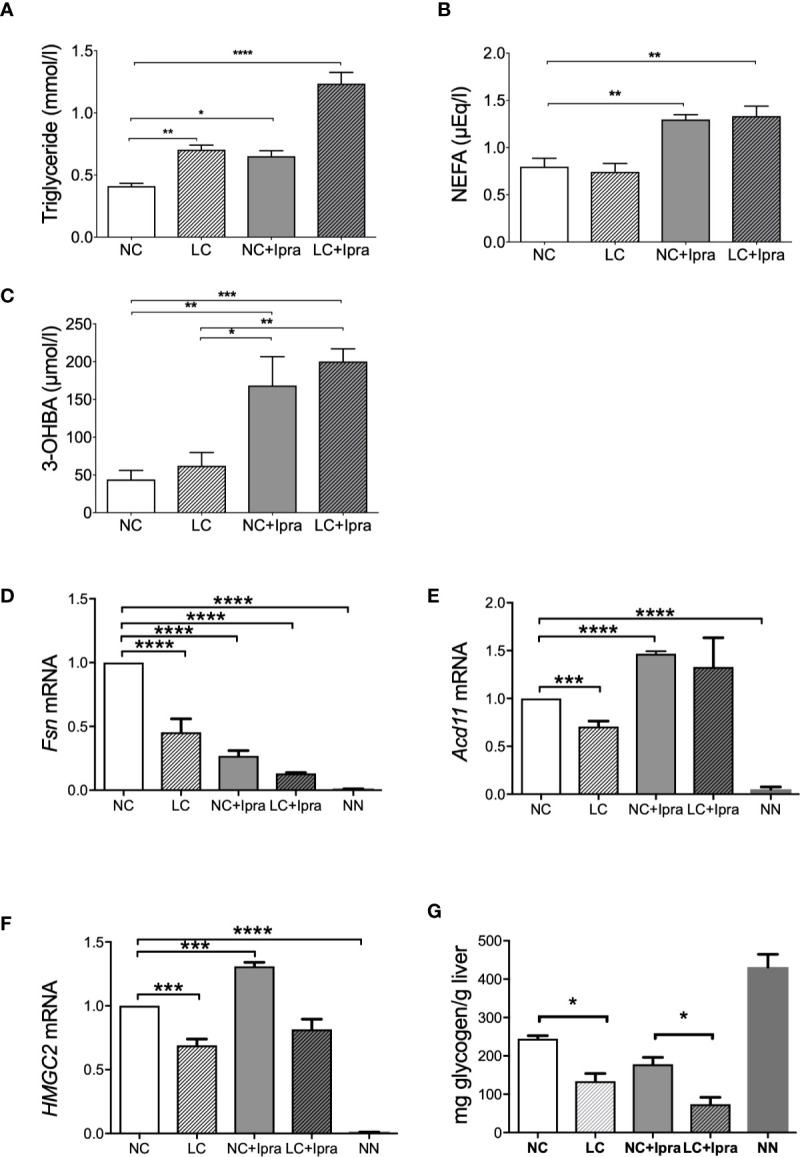
Lipid metabolism, ketogenesis, and glycogen content in the liver. Serum concentrations of **(A)** triglycerides (TG), **(B)** non-esterified fatty acid (NEFA), **(C)** 3-OHBA concentrations were shown. **(D)** *Fsn*, **(E)** *Acd11*, **(F)** *HMGC2* mRNA expressions in the liver. **(G)** Glycogen content in the liver. Data are presented as means ± SEM. n=6, each group. *p < 0.05, **p < 0.01, *** p < 0.001, ****p < 0.0001. White bars, normal-carbohydrate diet (NC); hatched white bars, low-carbohydrate diet (LC); grey bars, NC+Ipra; hatched gray bars, LC+Ipra. NN: non-diabetic mouse.

Lastly, we investigated fat, ketone, and glycogen metabolism in Akita mice during the treatments. Both LC and SGLT2i therapy increased and the combination therapy further raised triglyceride levels (**Figure 4A**). Free fatty acid levels were significantly elevated in the SGLT2i treated groups (**Figure 4B**). In contrast, the gene expression involved in fatty acid synthesis (*Fasn*) was inversely suppressed by both LC and SGLT2i, and by the combination (LC+Ipra) (**Figure 4D**). The gene expression involved in beta-oxidation (*Acd11)* was significantly diminished by LC, but potentiated by SGLT2i (**Figure 4E**).

Ketone production was approximately four-fold potentiated by SGLT2i in Akita mice, but LC treatment alone did not change 3-OHBA levels, compared to the non-treated NC animals. Besides, the combination did not induce further ketone production in comparison with SGLT2i (**Figure 4C**). The gene expression involved in ketogenesis in the liver (*HMGC2*) was enhanced by SGLT2i, but suppressed by LC with significance (**Figure 4F**).

Glycogen storage was diminished in the liver of Akita mice compared to non-diabetic NN animals. LC, but not SGLT2i, induced depletion of glycogen (**Figure 4G**).

## Discussion

Male Akita mice present lean and reduced beta-cell mass with diminished insulin secretion due to Ins2 gene mutation (14). We chose male Akita mice in the current study, since the Japanese population with diabetes is characterized by impaired insulin and relatively low BMI, compared to Western populations (15).

We demonstrated in the current study that both LC and SGLT2i prevented the progression of chronic hyperglycemia in Akita mice, while we did not observe casual glycemic change by those treatments in the non-diabetic mice (11). Initially, we had suspected that SGLT2i treatment with restriction of carbohydrate intake might be less effective or harmful especially along with LC, because of shifting to catabolic status. However, rather than the harms such as severe ketosis, the combination therapy showed a definite lowering of casual glycemia and HbA1c levels compared to the treatment of LC or SGLT2i alone.

Male Akita mice develop severe hyperglycemia accompanied by hyperphagia in the first several weeks after birth, in comparison with the female (16). Hyperphagia is a major factor, which can worsen chronic hyperglycemia in the diabetic state. We previously demonstrated that SGLT2i increased calorie intake in the non-diabetic mice (11). In human, SGLT2i strongly reduces body weight in the first several months, but this effect does not continue thereafter. SGLT2i can increase food intake and alter energy expenditure, resulting in deceleration of body weight reduction (17). In contrast, we observed that SGLT2i alone decreased and the combined therapy additionally diminished food intake, possibly rendering Akita mice to lower glycemia. It is obscure how both LC and SGLT2i exerted anorexic effect in Akita mice.

We observed that the combination therapy significantly ameliorated islet morphology representing enhanced beta/alpha cell area ratio, although we did not observe sufficient improvement of insulin secretion. In the islets, some studies have indicated that alpha-cells expressed SGLT2 and SGLT2i enhanced glucagon secretion (18–20). Others indicated that alpha-cells expressed SGLT1 but not SGLT2 (21, 22). However, a recent report indicated that SGLT2 was not expressed in both rodent and human pancreatic alpha- and beta-cells and concluded that SGLT2i did not directly modulate insulin and glucagon secretion (23). Our previous reports indicated that the improvement of the beta/alpha cell area ratio was crossly related to recovery from chronic hyperglycemia (12, 13). A non-selective SGLT inhibitor, phrolizin, preserved islet morphology in 90% pancreatectomized hyperglycemic rats *via* increasing GIP and GLP-1 receptor expressions (24). We speculated that relief from glucotoxicity by LC and SGLT2i could indirectly support to prevent beta-cell death and alpha-cell expansion in part. Some previous reports indicated that beta-cell mass was sustained by treatment with either SGLT2i alone or SGLT2i plus insulin, but not by insulin alone in diabetic rodent models (25–27). Thus, we speculate that SGLT2i per se might directly protect islet morphology. We had expected further protection by the treatments, but the protection was relatively limited. We speculate that was because beta-cell dysfunction is very severe and progressive due to advanced ER stress in Akita mice.

In the current study, gluconeogenic gene expressions were extremely enhanced both in the kidney and the liver of Akita mice compared to the tissues of the non-diabetic mice. We previously showed that G6PC, Pck, and Fbp gene expressions were simultaneously increased in the kidney by SGLT2i treatment, but in the liver by LC treatment in the non-diabetic mice (11). Akita mice are insulinopenic and insulin suppresses transcription of those gluconeogenic genes *via* FoxO1 nuclear localization (28). We speculate that gluconeogenesis was extremely enhanced in both the kidney and the liver, which could not be sufficiently modified by LC or SGLT2i.

SGLT2i, but not LC, increased production of ketone bodies in Akita mice. It was also notable that the combination did not enhance the further production of ketone bodies. Dehydration is a crucial player for pathogenesis of euglycemic ketoacidosis during the SGLT2i treatment in insulinopenic diabetes (29). In contrast, LC, but not SGLT2i, increased body weight and fat mass. The accurate mechanism is obscure, but we speculate that LC protected excess lipid utilization resulting in the maintenance of body weight and lowered ketogenesis in comparison to SGLT2i in Akita mice. Indeed, we observed that LC did not raise NEFA release with a reduction of lipolytic Acd11 gene and ketogenic HMGC2 gene expressions, in comparison to the non-treated Akita mice. We observed only HMGCS2 as a ketogenesis enzyme. But the levels of ketone bodies are controlled by the balance of production and consumption. SCOT is an enzyme of ketone oxidation to acetyl-CoA, therefore we should examine SCOT as well as another enzyme BDH1 expression in the future study. HMG-CoA lyase could be examined, since the enzyme is also involved in the ketone production.

It is still obscure how LC and SGLT2i treatments and combination treatment showed beneficial outcomes. We presume that increase of fat oxidation might be important through energy expenditure shift from carbohydrate to fat by SGLT2i. Indeed, Acs11 mRNA expression was enhanced by SGLT2i. But unexpectedly, the combination did not alter Acs11 expression. We presume LC might reduce hyperglycemia by direct reduction of sugar influx to the blood stream. But it is still possible that LC might enhance fat metabolism, though LC contains relatively high fat content. We need to consider another possibility that Acs11 protein levels could be enhanced by fat rich LC, through post-translational regulations. Thus, still further examinations should be performed until the mechanism is unveiled.

In the previous study, a low-carbohydrate and therefore high-fat diet increased body weight and adipose tissue mass but exacerbated glucose intolerance in prediabetic New Zealand obese mice (30). While, a very low carbohydrate ketogenic diet improves glucose tolerance in leptin-deficient mice independently of weight loss (31). We demonstrated here that LC could be beneficial to treat diabetes without obesity and hyperinsulinemia.

There are some limitations in the current study. First, we cannot assert that male Akita mice are an ideal model for human lean T2DM subjects. Indeed, Akita mice were primarily referred to as ‘a murine MODY model’, since they were non-obese and early-onset NIDDM (16, 32). They may present phenotypes associated with type 1 diabetes rather than T2DM, since they present severe beta-cell dysfunction with low insulin levels. We also note that sex-specific differences cannot be addressed in this study. Similarly to other animal diabetic models, there are different phenotypes by the sex in Akita mice. In fact, female Akita mice present mild hyperglycemia compared to the male counterpart. It was indicated that estrogen could prevent beta-cell apoptosis. It can be informative that we test the female counterpart in the future’s study. Second, our LC contains a relatively higher content of protein, which may influence appetite or renal excretion of protein. We need to consider our LC contains a relatively higher content of protein, which might induce abdominal fat accumulation. Third, we should have examined basal metabolism, respiratory quotient, and changes in body temperature to elucidate the mechanism of body weight. Lastly, we should have conducted another study using Akita mice under paired-feeding to exclude influences of calorie consumption and to evaluate the essential effects of LC and SGLT2i. In addition, we must note again that the combination of LC and SGLT2i can induce ketoacidosis in the subjects of insulin-deficient T1DM with uncontrolled insulin injection in the clinical settings.

In conclusion, we observed that both LC and SGLT2i reduced chronic hyperglycemia and the combination presented synergistic favorable effects in Akita mice. SGLT2i, but not LC, enhanced ketogenesis, while the combination did not induce further ketosis. However, additional studies are necessary to investigate the mechanism in detail.

## Data Availability Statement

The original contributions presented in the study are included in the article/**Supplementary Material**. Further inquiries can be directed to the corresponding author.

## Ethics Statement

The animal study was reviewed and approved by Asahikawa Medical University Animal Research Committee (No15070, No14063, and No16129).

## Author Contributions

YF, KA, YT, TY, YM, and MH contributed to the study concept and design. YF, KA, and YT conducted research;. YF, KA, YT, TY, YM, and MH analyzed and interpreted the data and performed statistical analysis. YF, KA, and YT drafted the manuscript. YF, KA, YT, TY, YM, and MH reviewed the manuscript for important intellectual content. YF had primary responsibility for final content. All authors contributed to the article and approved the submitted version.

## Conflict of Interest

The authors declare that the research was conducted in the absence of any commercial or financial relationships that could be construed as a potential conflict of interest.

## Abbreviations

HbA1c, glycated hemoglobin; 3-OHBA, hydroxybutyrate; LC, low-carbohydrate diet; NEFA, non-esterified fatty acid; NC, normal-carbohydrate diet; PBS, phosphate-buffered saline; SGLT2i, sodium-glucose cotransporter 2 inhibitor; TG, triglyceride, TG; T1DM, type 1 diabetes mellitus; T2DM, type 2 diabetes mellitus; MODY, maturity onset diabetes of the young; NIDDM, Non-insulin dependent diabetes mellitus.
