# Supplementary material for: A Low-Carbohydrate Diet Improves Glucose Metabolism in Lean Insulinopenic Akita Mice Along With Sodium-Glucose Cotransporter 2 Inhibitor
Source: Front Endocrinol (Lausanne). 2020 Dec 11;11:601594. doi: 10.3389/fendo.2020.601594 (PMC7759607; doi:10.3389/fendo.2020.601594)
Supplement: Supplementary file 1 [file DataSheet_1.docx]

Supplementary Material

## Supplementary Figures


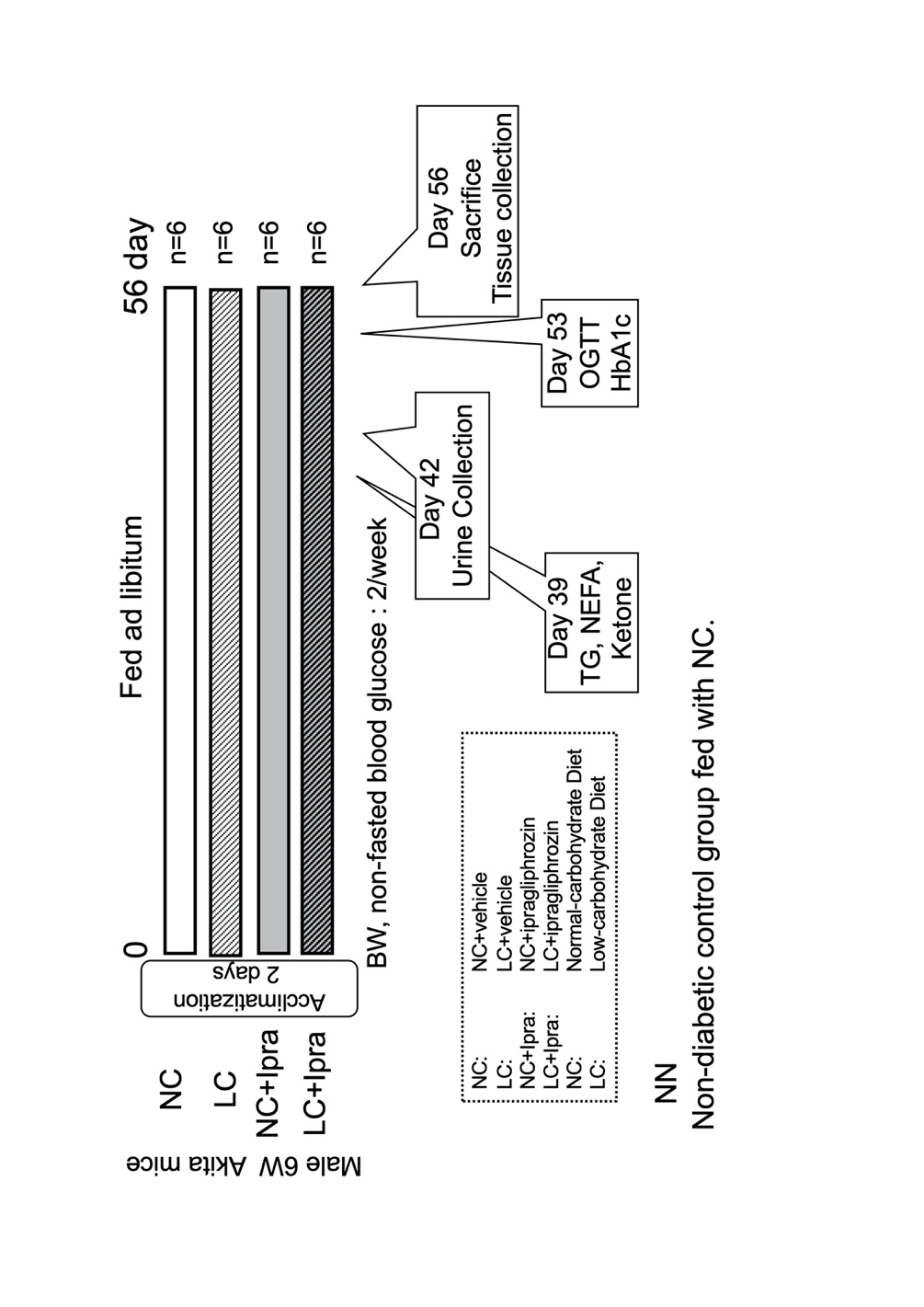


**Supplementary Figure 1.** The experimental Protocol.
